# Supplementary material for: Memory-electroluminescence for multiple action-potentials combination in bio-inspired afferent nerves
Source: Nat Commun. 2024 Apr 25;15:3505. doi: 10.1038/s41467-024-47641-6 (PMC11045776; doi:10.1038/s41467-024-47641-6)
Supplement: Supplementary file 1 — Supplementary Information [file 41467_2024_47641_MOESM1_ESM.pdf]

# Supplementary Information

## Memory-electroluminescence for multiple action-potentials combination in bio-inspired afferent nerves

Kun Wang[1], Yitao Liao[1], Wenhao Li[1], Junlong Li[1], Hao Su[1], Rong Chen[2], Jae Hyeon Park[3], Yongai Zhang[1,2], Xiongtu Zhou[1,2], Chaoxing Wu[1,2*], Zhiqiang Liu[4*], Tailiang Guo[1,2*], Tae Whan Kim[3*]

[1] College of Physics and Information Engineering, Fuzhou University, Fuzhou 350108, China.

[2] Fujian Science & Technology Innovation Laboratory for Optoelectronic Information of China, Fuzhou 350108, China.

[3] Department of Electronic and Computer Engineering, Hanyang University, Seoul 133-791, Korea

[4] Research and Development Center for Semiconductor Lighting Technology, Institute of Semiconductors, Chinese Academy of Sciences, Beijing 100083, China.

[*] Authors to whom correspondence should be addressed.

Email addresses: chaoxing_wu@fzu.edu.cn (C. Wu), lzq@semi.ac.cn (Z. Liu), gtl_fzu@hotmail.com (T. Guo), twk@hanyang.ac.kr (T. W. Kim)

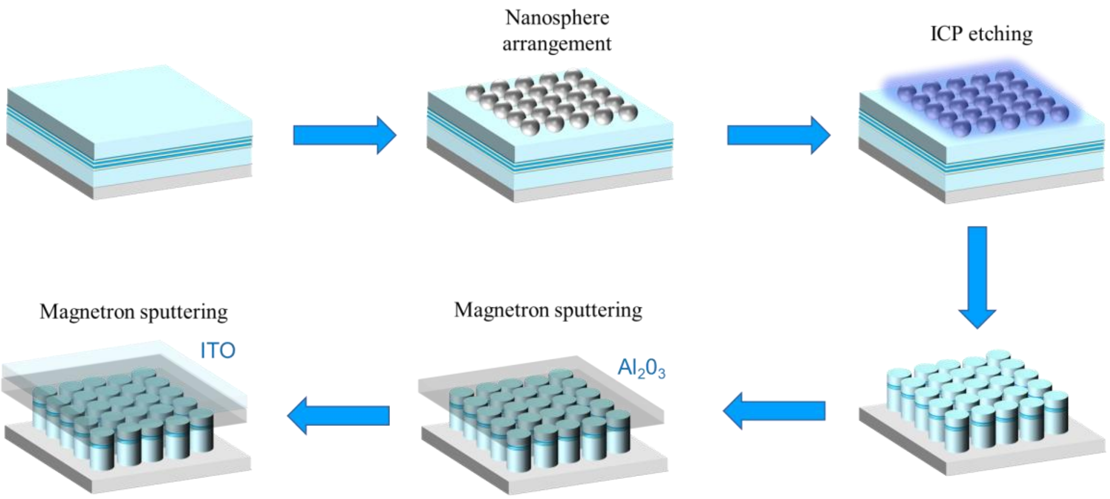


**Supplementary Figure 1.** Fabrication of Nano-LEDs. $SiO_2$ nanospheres (~600 nm in diameter) are used as masks. Inductively coupled plasma etching is used to etch the blue LED wafer. $Al_2O_3$ is deposited on the Nano-LED to a thickness of 60 nm, followed by deposition of a 100-nm-thick ITO electrode on the insulating layer by using magnetron sputtering.

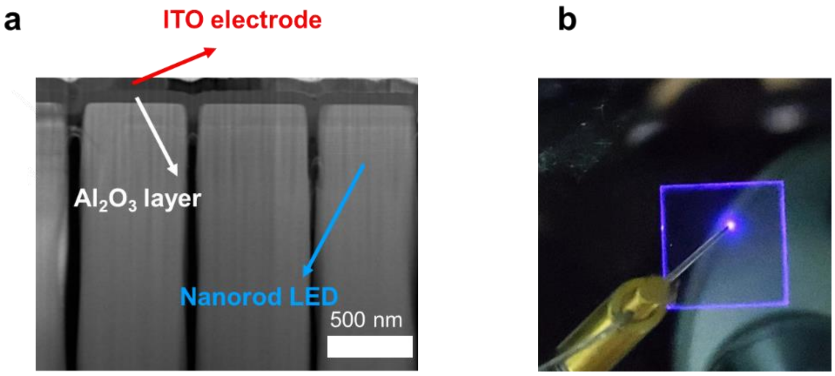


**Supplementary Figure 2.** (a) Scanning transmission electron microscope image and (b) actual photo of the Nano-LED in operation.

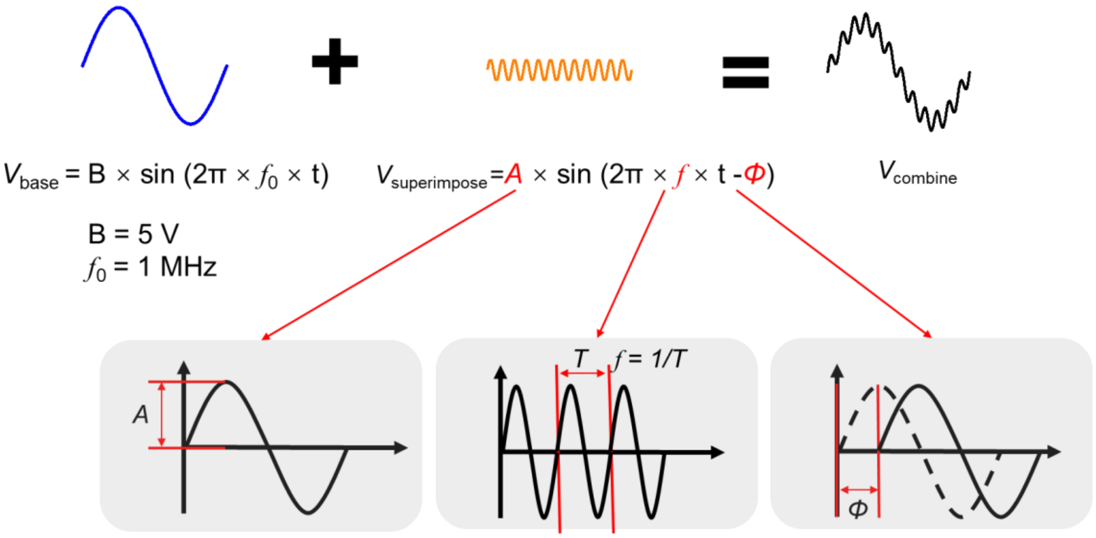


**Supplementary Figure 3.** Construction of $V_{combine}$, and the amplitude ($A$), frequency ($f$), and phase difference ($\Phi$) of $V_{superimpose}$.

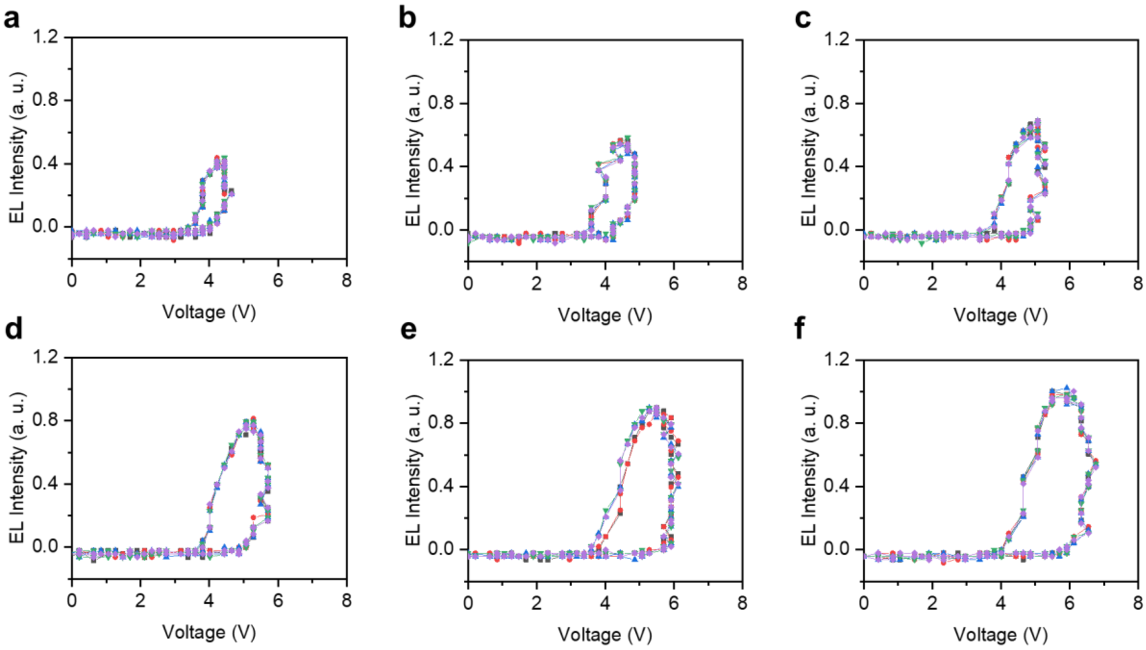


**Supplementary Figure 4.** EL intensity-voltage relationship with increasing amplitude of the AC voltage. The amplitudes of the voltages applied to the device are (a) 4.65 V, (b) 4.85 V, (c) 5.30 V, (d) 5.70 V, (e) 6.15 V, and (f) 6.75.

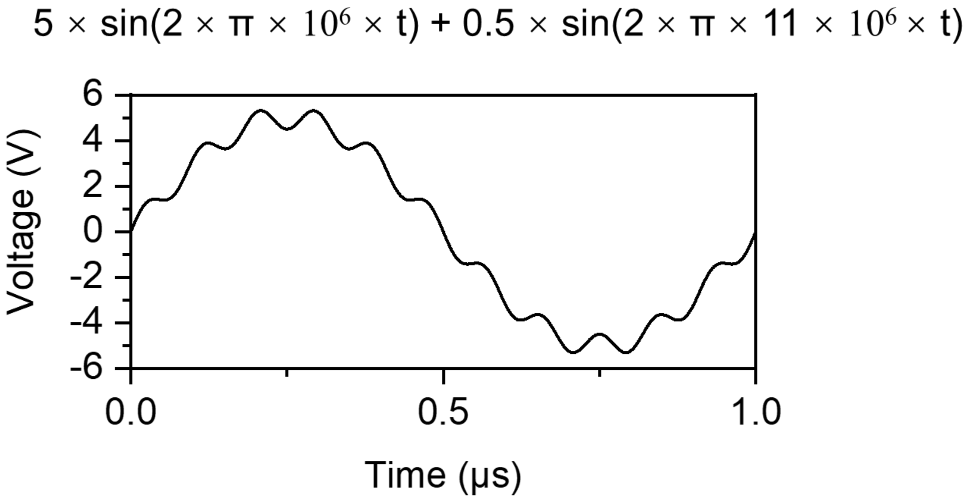


**Supplementary Figure 5.** $V_{combine}$ used in the simulation of the Nano-LED.

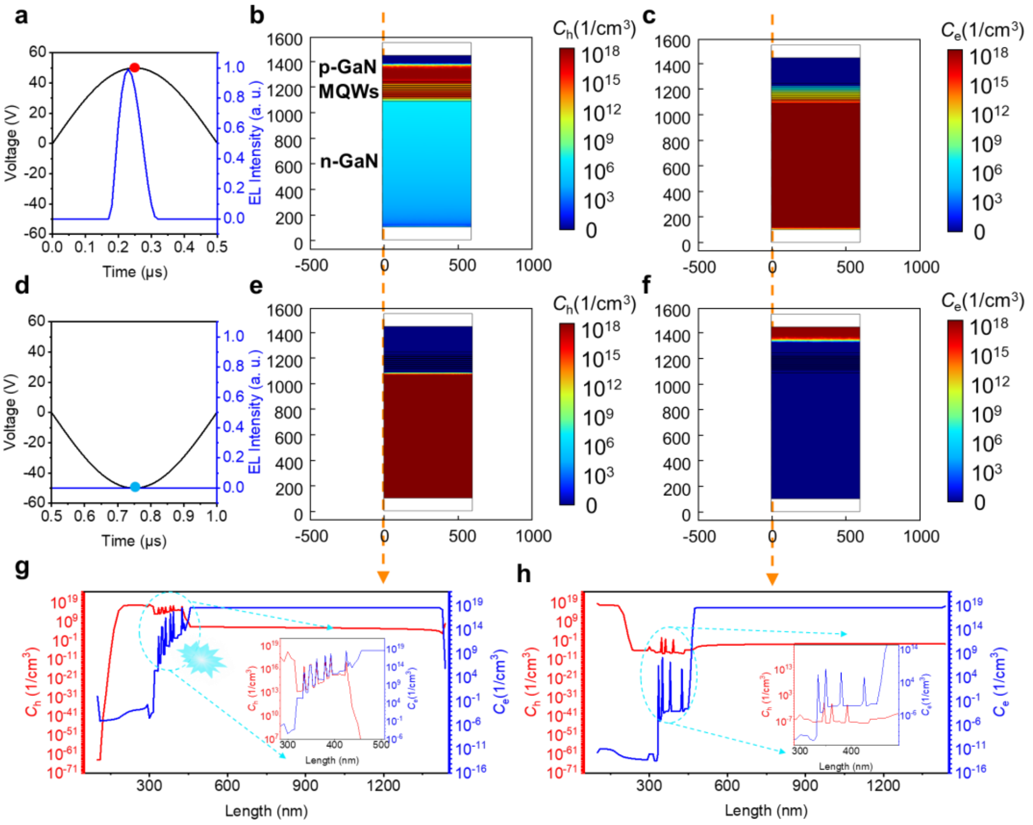


**Supplementary Figure 6.** Finite element analysis (FEA) of the 2D modeled Nano-LED. (a) EL is generated in the positive half cycle of the sinusoidal voltage. Red point: the moment when the sinusoidal voltage signal reaches its maximum. (b) Hole and (c) electron concentration redistributions at that moment in Supplementary Fig. 6a. (d) No EL is generated in the nagetive half cycle of the sinusoidal voltage. Blue point: the moment when the sinusoidal signal voltage reaches its minimum. (e) Hole and (f) electron concentration redistributions at the moment in Supplementary Fig. 6d. (g) Carrier concertrations along the dotted lines in Supplementary Figs. 6b and 6e. (h) Carrier concertrations along the dotted lines in Supplementary Figs. 6c and 6f.

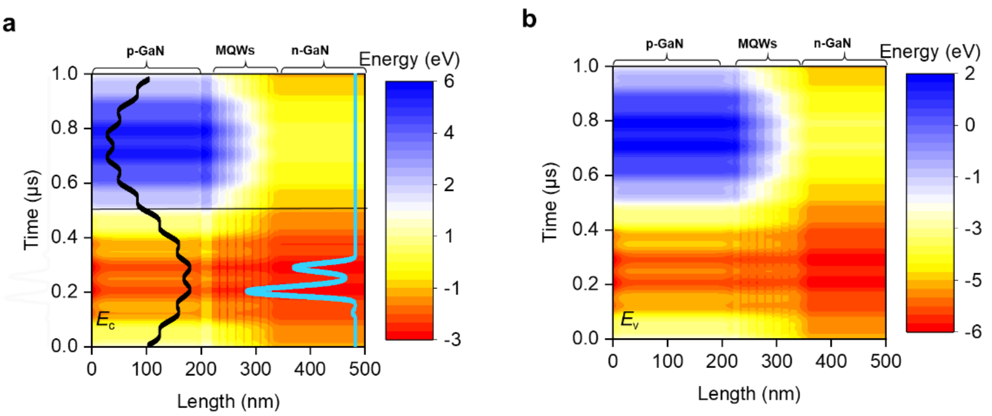


**Supplementary Figure 7.** Heat maps of variations in the energy band of the Nano-LED. (a) Conduction band ($E_c$) and (b) valence band ($E_v$) near the MQWs during the cycle of $V_{combine}$. The waveform represented by the blue line is the EL while the one depicted by the black line is $V_{combine}$.

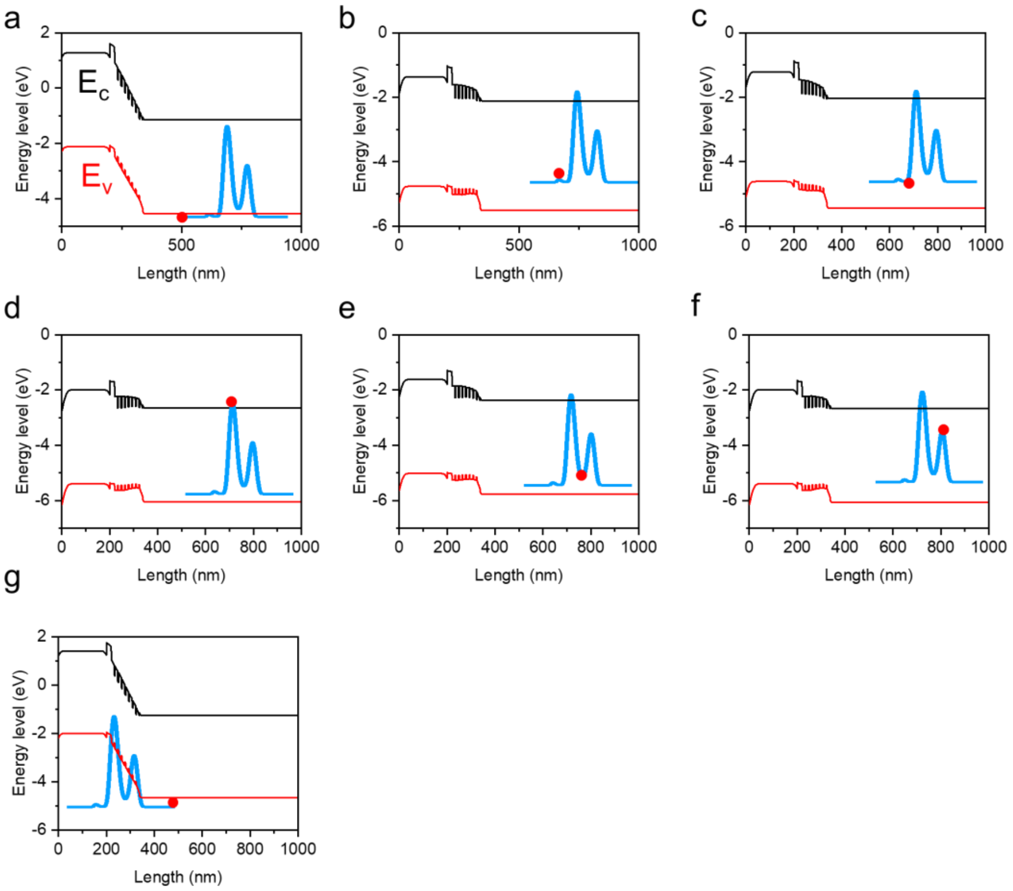


**Supplementary Figure 8.** Energy bands in the MQWs (black lines: $E_C$, red lines: $E_V$) at seven different moments (red point in the inset) during the positive half cycle. (a) Initial state with no voltage applied. (b) As the Voltage increases, the barrier is low enough, and the first radiative recombination occurs. (c) The barrier increases when the voltage decreases due to $V_{superimpose}$, and the first EL spike disappears. (d) When the voltage increases due to the $V_{superimpose}$, the barrier decreases again, and the second radiative recombination occurs. (e) (f) The third radiative recombination occurs when another fluctuating voltage (first decreases and then increases) is applied. (g) When the positive half-cycle of the voltage ends, the barrier returns to a higher value, stopping the radiation recombination.

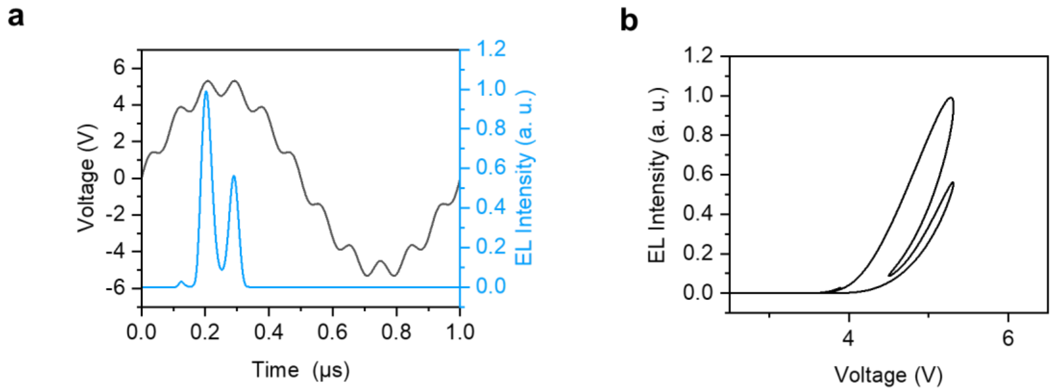


**Supplementary Figure 9.** (a) Waveform of a simulated Mem-EL spike and (b) EL intensity-voltage curve for the Nano-LED driven by $V_{\mathrm{combine}}$.

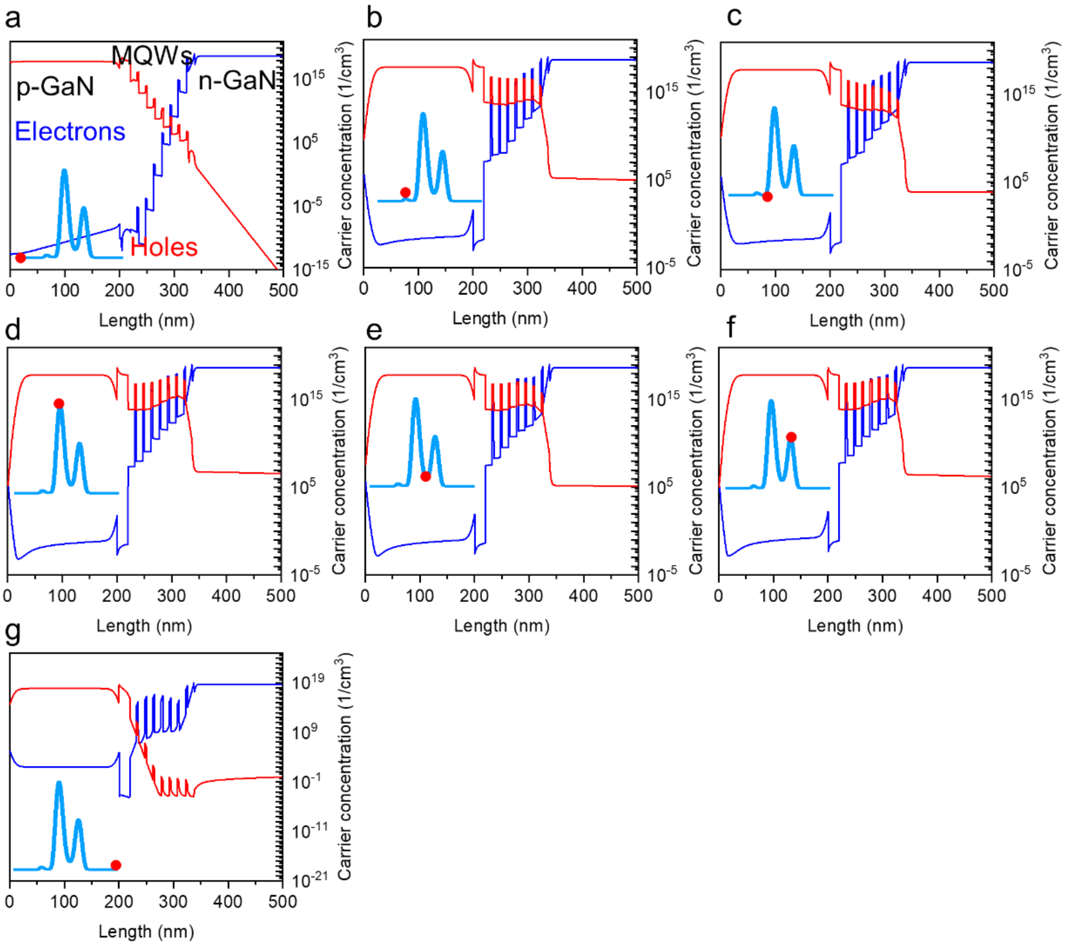


**Supplementary Figure 10.** Simulated carrier redistribution (blue line: electrons, red line: holes) near MQWs at seven different moments (red point in the inset) during the positive half cycle. (a) In the initial state, no radiative recombination occurs inside the MQWs without signal driving. (b) With increasing $V_{\text{combine}}$, the first radiative recombination occurs in the MQWs under the applied electric field. (c) The first radiative recombination is stopped or attenuated. (d) On the contrary, when $V_{\text{combine}}$ rises due to the application of $V_{\text{superimpose}}$, the hole concentration in the MQWs increases, and the second EL spike occurs. (e) (f) Similarly, when another fluctuating voltage (first decreases and then increases) is applied to the device, the hole concentration in the MQWs first decreases and then increases, which causes the third EL spike. (g) When the positive half-cycle of the driving voltage ends, the hole concentration in the p-region and the electron concentration in the n-region return to higher values.

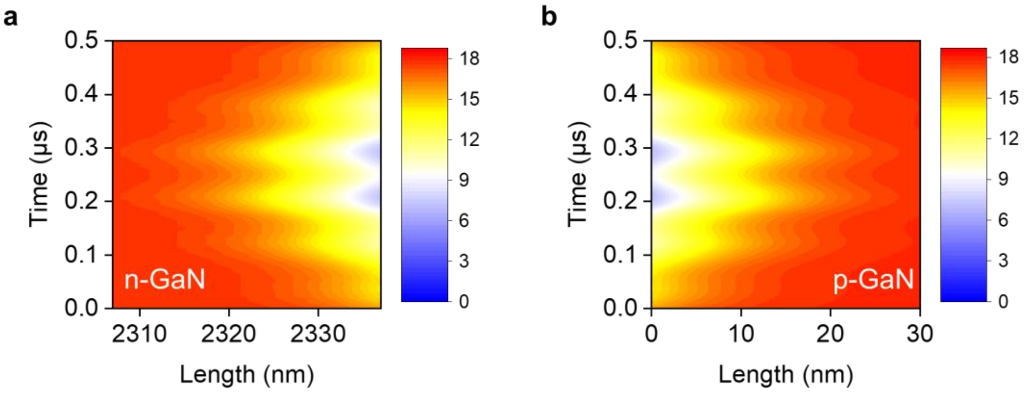


**Supplementary Figure 11.** (a) Electron concentration redistribution in n-GaN and (b) hole concentration redistribution in p-GaN for the same doping concentrations.

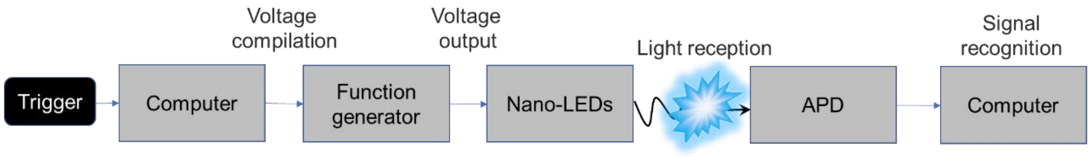


**Supplementary Figure 12.** Flow chart of the artificial brain-recognition system based on the Nano-LEDs afferent nerve.

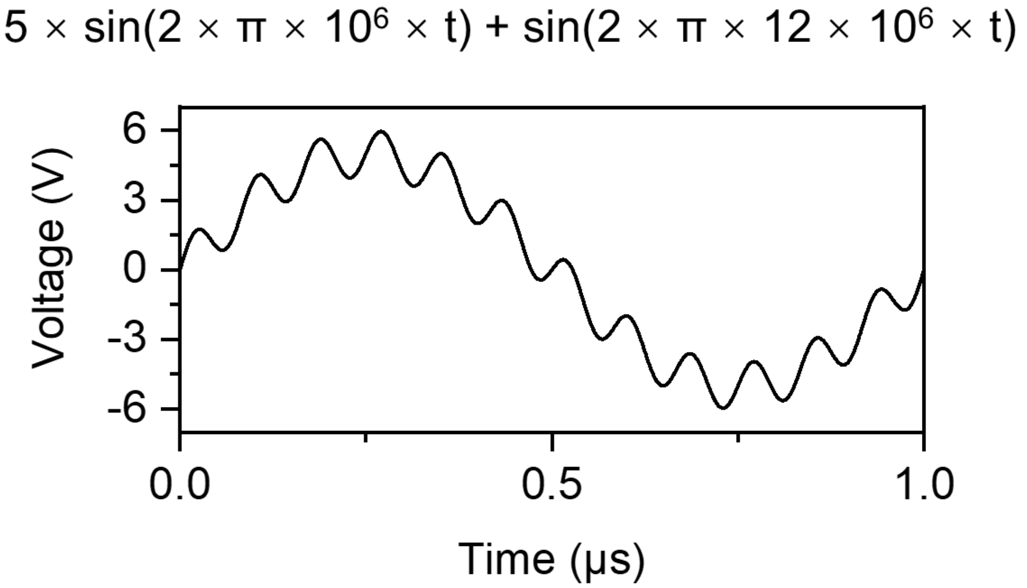


**Supplementary Figure 13.** $V_{combine}$ obtained after the encoding and mapping of sensor 'S104'.

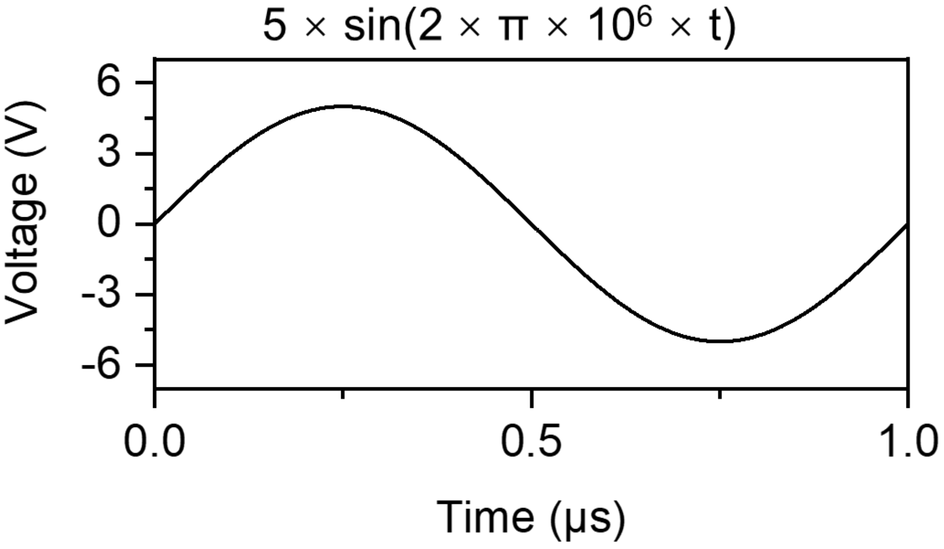


**Supplementary Figure 14.** Fixed $V_{\text{base}}$ used in the experiment and simulation.

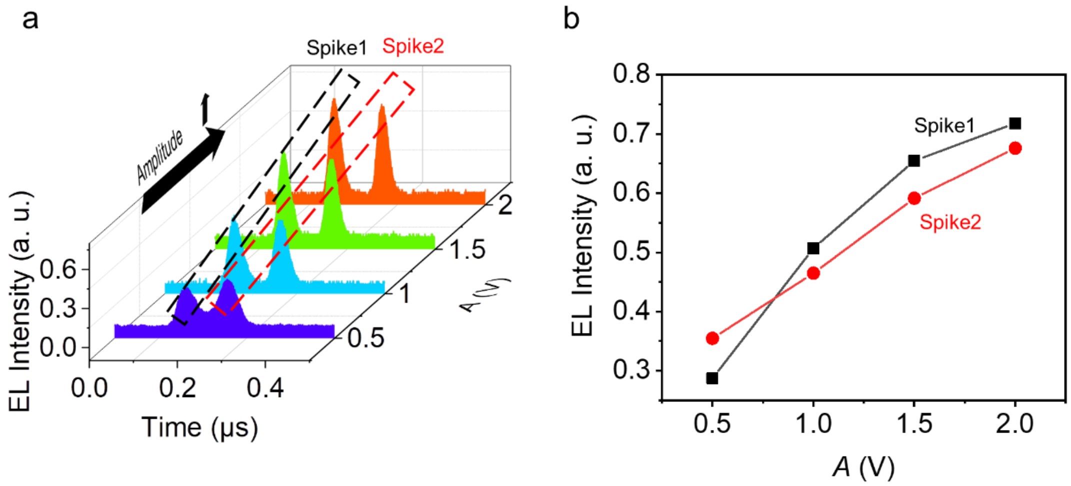


**Supplementary Figure 15.** (a) Waveforms of the two EL spikes and (b) variations in the peak EL-intensity of the two EL spikes (black line: spike 1, red line: spike 2) as the amplitude ($A$) of $V_{\text{superimpose}}$ increases.

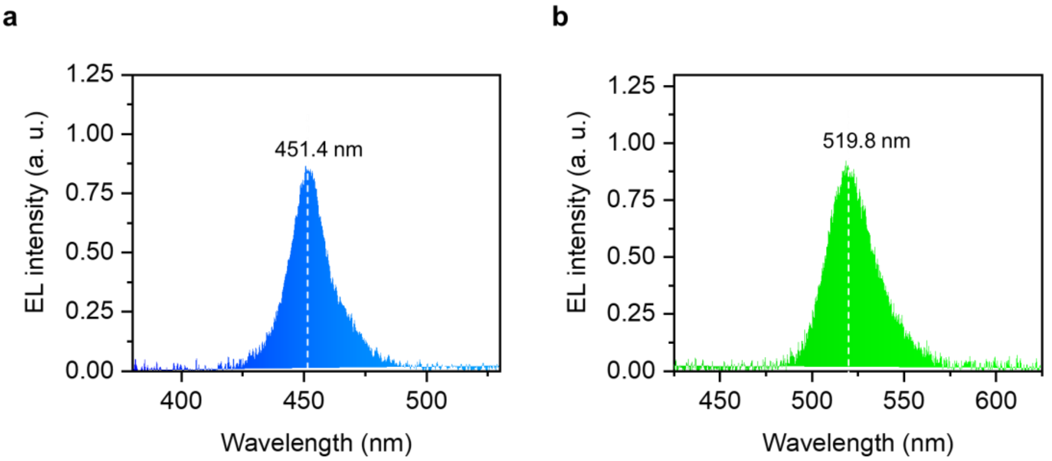


**Supplementary Figure 16.** The spectra of (a) the blue and (b) the green device.

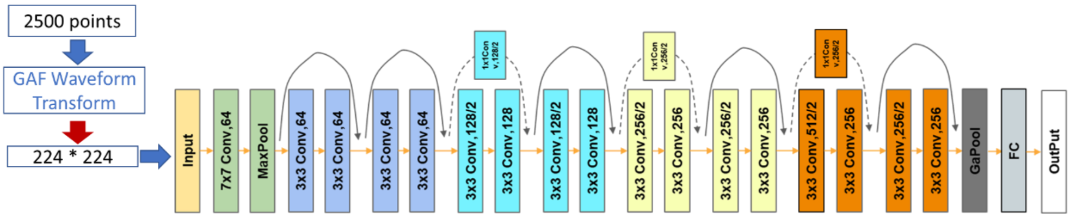


**Supplementary Figure 17.** Structure of the GAF-ResNet. Each waveform consists of 2500 points. After the GAF transformation and a series of convolutional and pooling processes, 256 features are extracted and then sent to the full-connect layer. Finally, 256 classifications are calculated.

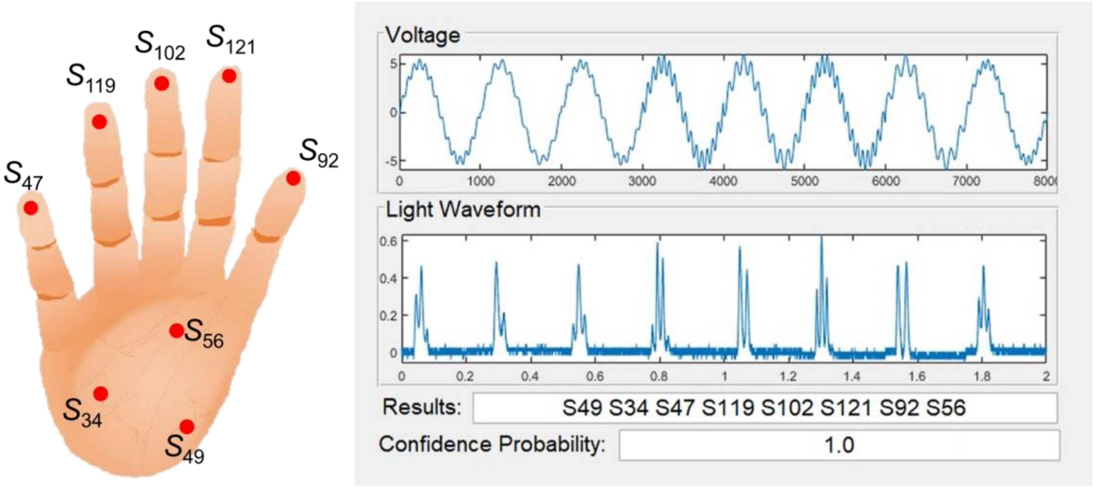


**Supplementary Figure 18.** Software interface of the sensor position recognition system based on the Nano-LED, including sensors distributed in the palm, the voltage signal interface, the light waveform interface, output results, and the real-time confidence probability. When a sensor is triggered, a corresponding $V_{\text{combine}}$ signal is generated to drive the Nano-LED to emit Mem-EL spikes. The Mem-EL spikes are fed into the neural network to identify the triggered output sensor number.

**Supplementary Table 1**

Parameters of the simulated Nano-LED structure.

| | Band gap /V | Electron affinity /V | Electron mobility /[cm^2/(V*s)] | Hole mobility /[cm^2/(V*s)] | Doping concentration /(1/cm^3) |
|---|---|---|---|---|---|
| p-GaN | 3.4 | 4.1 | 55 | 10 | $7e^{17}$ |
| n-GaN | 3.4 | 4.1 | 200 | 3 | $5e^{18}$ |
| $In_{0.01}Ga_{0.99}N$ | 3.35884 | 4.12881 | 685 | 153.3 | / |
| $In_{0.02}Ga_{0.98}N$ | 3.31797 | 4.15742 | 685 | 153.3 | / |
| $In_{0.03}Ga_{0.97}N$ | 3.27739 | 4.18583 | 685 | 153.3 | / |
| $In_{0.04}Ga_{0.96}N$ | 3.23709 | 4.21404 | 685 | 153.3 | / |
| $In_{0.05}Ga_{0.95}N$ | 3.19708 | 4.24205 | 685 | 153.3 | / |
| $In_{0.15}Ga_{0.85}N$ | 2.81268 | 4.51113 | 685 | 153.3 | / |
| $Al_{0.15}Ga_{0.85}N$ | 3.693 | 3.575 | 55 | 10 | $7e^{17}$ |

**Supplementary Table 2**

Mapping relationship between the sensors (0-255) and the amplitude (*A*), the frequency (*f*) , and the intensity (*Φ*) of $V_{superimpose}$.

| | **Binary coding** | ***A*** | ***f*** | ***Φ*** |
|---|---|---|---|---|
| Sensor 0 | 00000000 | 0.5 V | 2 MHz | 0° |
| Sensor 1 | 00000001 | 0.5 V | 2 MHz | 90° |
| Sensor 2 | 00000010 | 0.5 V | 2 MHz | 180° |
| Sensor 3 | 00000011 | 0.5 V | 2 MHz | 270° |
| Sensor 4 | 00000100 | 0.5 V | 3 MHz | 0° |
| … | | … | … | … |
| Sensor 64 | 0100000 | 1 V | 2 MHz | 0° |
| …. | | …. | …. | …. |
| Sensor 254 | 11111110 | 2 V | 17 MHz | 180° |
| Sensor 255 | 11111111 | 2 V | 17 MHz | 270° |

**Supplementary Note 1**

To further verify the above process, we used the FEA method to simulate a 2D-modeled Nano-LED working in the non-carrier injection mode. The redistributions of the carrier concentrations in the positive and the negative half-cycles are shown in Supplementary Fig. 6. During the positive half-cycle, the voltage reaches its maximum (Supplementary Fig. 6a), and the holes in the p-region and the electrons in the n-region are driven into the MQWs under the external electric field. The electron-hole concentrations in the MQW are high, so radiative recombination occurs. An approximately 50-nm-thick region at the top of the p-GaN is depleted, resulting in a very low hole concentration, as shown in Supplementary Fig. 6b. Similarly, due to the insulating layer between the n-GaN and the external electrodes, a depletion region also exists at the bottom of the n-GaN. Therefore, the electron concentration decreases at the bottom of n-GaN, as shown in Supplementary Fig. 6c.

At the moment the negative half-cycle voltage reaches its minimum (Supplementary Fig. 6d), the depletion regions at the top of p-GaN and the bottom of n-GaN disappear, and the hole concentration in the p-region and the electron concentration in the n-region recover to higher values. Moreover, the carrier concentrations in the MQWs decrease without radiative recombination, as shown in Supplementary Figs. 6e and 6f. The carrier concentrations along the dashed lines are provided to show the details. As Supplementary Fig. 6g shows, when the voltage reaches its maximum, both the p- and the n-regions show depleted states, and the

carrier concentrations in the MQWs reach high levels. However, when the voltage reaches its minimum, the carrier concentrations in the MQWs are insufficient for radiation recombination to occur, and sufficient carriers accumulate in the p- and the n-regions for radiative combination to occur during the next positive half-cycle

According to the above discussion, the applied negative half-cycle voltage causes the charge accumulated during the previous positive half-cycle voltage to be released, allowing the internal carriers of the LED to be restored to their initial states or sufficient carriers to accumulate for radiative recombination during the next positive half-cycle. Therefore, the negative half-cycle voltage cannot be ignored.
