# Supplementary material for: Memory-electroluminescence for multiple action-potentials combination in bio-inspired afferent nerves
Source: Nat Commun. 2024 Apr 25;15:3505. doi: 10.1038/s41467-024-47641-6 (PMC11045776; doi:10.1038/s41467-024-47641-6)
Supplement: Supplementary file 3 — Description of additional supplementary files [file 41467_2024_47641_MOESM3_ESM.pdf]

DESCRIPTION OF ADDITIONAL SUPPLEMENTARY FILES DOCUMENT

Supplementary Movie 1

Legend: A video demonstration of the EL Intensity-Voltage relationship in traditional carrier injection mode.

Supplementary Movie 2

Legend: A video demonstration of the EL Intensity-Voltage relationship in non-carrier injection mode.

Supplementary Movie 3

Legend: A video demonstration of the EL Intensity-Voltage relationships in non-carrier injection mode varies with combined driving signals.

Supplementary Movie 4

Legend: A video demonstration of position recognition of sensors on the palm based on artificial afferent nerves.
